# Characterization of a migrative subpopulation of adult human nasoseptal chondrocytes with progenitor cell features and their potential for in vivo cartilage regeneration strategies

**DOI:** 10.1186/s13578-016-0078-6

**Published:** 2016-02-13

**Authors:** A. F. Elsaesser, S. Schwarz, H. Joos, L. Koerber, R. E. Brenner, N. Rotter

**Affiliations:** Department of Oto-Rhino-Laryngology, Head and Neck Surgery, Ulm University Medical Center, Frauensteige 12, 89075 Ulm, Germany; Department of Orthopedics, Division for Biochemistry of Joint and Connective Tissue Diseases, University of Ulm, Ulm, Germany; Department of Chemical and Biological Engineering, Institute of Bioprocess Engineering, University of Erlangen, Erlangen, Germany

**Keywords:** Migrative chondrocyte subpopulation, Chondrogenic progenitor cells, Mesenchymal stem cells, Human nasal septum, Decellularized extracellular matrix, Tissue engineering, Migration

## Abstract

**Background:**

Progenitor cells display interesting features for tissue repair and reconstruction. In the last years, such cells have been identified in different cartilage types. In this study, we isolated a migrative subpopulation of adult human nasoseptal chondrocytes with progenitor cell features by outgrowth from human nasal septum cartilage. These putative progenitor cells were comparatively characterized with mesenchymal stem cells (MSC) and human nasal septum chondrocytes with respect to their cellular characteristics as well as surface marker profile using flow cytometric analyses. Differentiation capacity was evaluated on protein and gene expression levels.

**Results:**

The migrative subpopulation differentiated into osteogenic and chondrogenic lineages with distinct differences to chondrocytes and MSC. Cells of the migrative subpopulation showed an intermediate surface marker profile positioned between MSC and chondrocytes. Significant differences were found for CD9, CD29, CD44, CD90, CD105 and CD106. The cells possessed a high migratory ability in a Boyden chamber assay and responded to chemotactic stimulation. To evaluate their potential use in tissue engineering applications, a decellularized septal cartilage matrix was either seeded with cells from the migrative subpopulation or chondrocytes. Matrix production was demonstrated immunohistochemically and verified on gene expression level. Along with secretion of matrix metalloproteinases, cells of the migrative subpopulation migrated faster into the collagen matrix than chondrocytes, while synthesis of cartilage specific matrix was comparable.

**Conclusions:**

Cells of the migrative subpopulation, due to their migratory characteristics, are a potential cell source for in vivo regeneration of nasal cartilage. The in vivo mobilization of nasal cartilage progenitor cells is envisioned to be the basis for in situ tissue engineering procedures, aiming at the use of unseeded biomaterials which are able to recruit local progenitor cells for cartilage regeneration.

## Background

Structural and functional defects of cartilage tissues in the head and neck region are frequently congenital, but also caused by trauma or cancer resections [1]. Since mature hyaline cartilage, as it is present in nasal septum, larynx and articular joints, has only limited regeneration capacity [2], defect reconstruction of craniofacial cartilage structures is based on the use of implant materials or autologous donor transplants [3–6]. In current reconstructive techniques, such as nasal reconstruction procedures, septal cartilage together with auricular and rib cartilage is widely used as gold standard for reconstructive surgery [7, 8]. In nasal reconstructive surgery nasal septal cartilage is preferred as tissue source when available owing to several important advantages such as suitable mechanical characteristics compared to e.g. costal and auricular cartilage [9].

These conventional reconstructions often involve additional surgical procedures which can be complicated by wound infections, insufficient cosmetic results and postoperative pain at the donor site. Due to the limited availability of donor cartilage tissue and marginal capacity for self-regeneration, cartilage tissue engineering rapidly developed in recent years to generate and promote applications in craniofacial reconstruction and facial plastic surgery [9, 10]. Thereby, the intention of cartilage tissue engineering is to develop and culture in vitro generated cartilage with structural, biochemical and functional properties comparable to native cartilage [11, 12] by combining in vitro engineered autologous cells and one or several suitable resorbable biomaterials [13]. The choice of the appropriate cell type for cell based tissue engineering strategies is a critical step as chondrocytes are differentiated and highly specialized cells responsible for the production of biomechanically appropriate extracellular matrix (ECM) of cartilage.

However, the chondrogenic potential of chondrocytes is limited as a consequence of proliferation [14] and represents a serious problem for the required extensive augmentation of cells, which is necessary to achieve sufficient implant sizes for clinical application [9, 14].

Mesenchymal stem cells (MSC) have been proposed as an attractive alternative cell source for cartilage tissue engineering [15, 16]. MSC can be expanded in vitro through several passages without loss of multipotentiality and phenotype [17]. As MSC are assumed to be involved in repair processes, they are accounted to be a useful cell source for creating regenerative autologous tissues [15, 18]. In the presence of specific chondrogenic stimulation, MSC can differentiate into chondrocytes generating cartilage specific ECM [15, 17]. Nevertheless, their potential use in cartilage tissue engineering is limited, as MSC seeded on collagen matrices produce less ECM components with inferior biomechanical stability as opposed to differentiated chondrocytes [19, 20].

MSC and local progenitor cells reside in bone marrow [21] and can also be easily obtained from various other adult mesenchymal and connective tissues such as synovium [22, 23] periosteum [24, 25] or adipose tissue [26].

Progenitor cells with chondrogenic origin have been isolated by enzymatic digestion of normal and osteoarthritic cartilage from human articular origin [27–29]. Recently, studies isolated chondrogenic progenitor cells (CPC) by outgrowth culture of cartilage fragments [30, 31]. Due to their migratory properties, these CPC were able to repopulate damaged tissue in vitro [31]. CPC from human articular cartilage chemotactically respond to growth factors such as platelet-derived growth factor (PDGF)-BB and insulin-like growth factor 1 (IGF-1) [30] in a comparable manner to mesenchymal progenitor cells from the bone marrow [32–34]. Based on these results together with our recent observation of cartilage regeneration in the rabbit nasal septum at the contact site between a decellularized collagen matrix and native septal cartilage (unpublished data), we hypothesized that human nasal cartilage also harbors a distinct subpopulation of migratory chondrocytes with progenitor cell features (mnCPC). Such cells could be a potential target for in situ nasal cartilage regeneration strategies.

Therefore the aims of the current study were (1) to establish outgrowth cultures of nasal septum cartilage and (2) to comparatively analyze characteristics of this cell population together with bone-marrow derived MSC and human nasal septum chondrocytes (hCh) with respect to their growth characteristics, surface marker profile expression, migratory activity and differentiation capacity. Third, the characteristics of these cells were compared to fully differentiated hCh in a model of in vitro tissue engineering using a decellularized extracellular cartilage matrix (DECM) [35] as a carrier.

## Results

HCh were isolated using enzymatic digestion with 0.3 % collagenase overnight. To isolate mnCPC cartilage fragments were placed into cell culture flasks and cells were allowed to actively migrate out of the cartilage fragments on the tissue culture plastic. The active migration was used as a selection criterion to yield mnCPC.

### Morphological and expansion characteristics

**Fig. 1:**
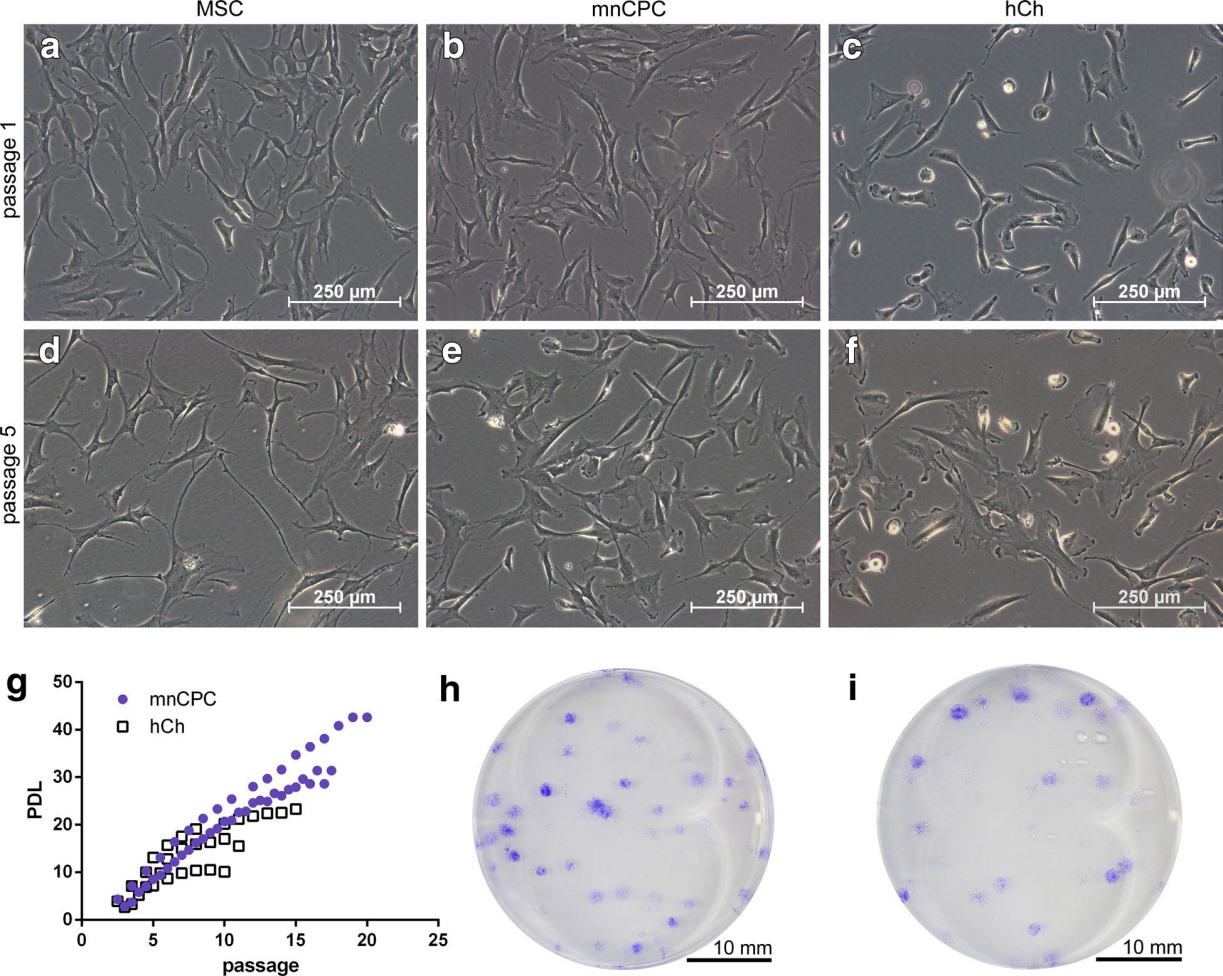
Expansion characteristics after passage 1 (**a**–**c**) and passage 5 (**d**–**f**). MnCPC proliferated up to 20 passages (**g**). Colony formation of mnCPC (**h**) and hCh (**i**) in 35 mm wells stained with *crystal violet* after 8 days. Representative images were chosen

MnCPC exhibited a flat, spindle-like and polygonal shape in passage 1 similar to MSC (Fig. 1a, b). HCh in contrast were smaller, rounded and less elongate (Fig. 1c). In passage 5 aging MSC developed thinner spindle-like cell filopodia and lost their shape while becoming more flat polygonal (Fig. 1d). MnCPC showed a comparable cell morphology, however, they did not lose their shape to the same extent (Fig. 1e). HCh kept their original round morphology until passage 5 (Fig. 1f).

HCh stopped their proliferation at passage 12 in average. In contrast, it was possible to culture mnCPC up to 20 passages (Fig. 1g).

The colony forming efficiency (CFE) was determined for mnCPC and hCh at a cell density of 100 cells per well initially seeded. 51.4 ± 7.47 % of the seeded mnCPC were able to form detectable colonies (Fig. 1h). In contrast, hCh formed significantly less colonies (40.0 ± 6.1 %, Fig. 1i).

### MnCPC express surface markers which share specific similarities with MSC and hCh

FACS analyses evidenced the complete absence of the hematopoietic stem cell markers CD34, CD133/1 as well as CD133/2 and the lymphocyte marker CD45 on mnCPC, hCh and MSC. Additionally, the endothelial cell marker CD31 was not detected in any of the cells.

**Fig. 2:**
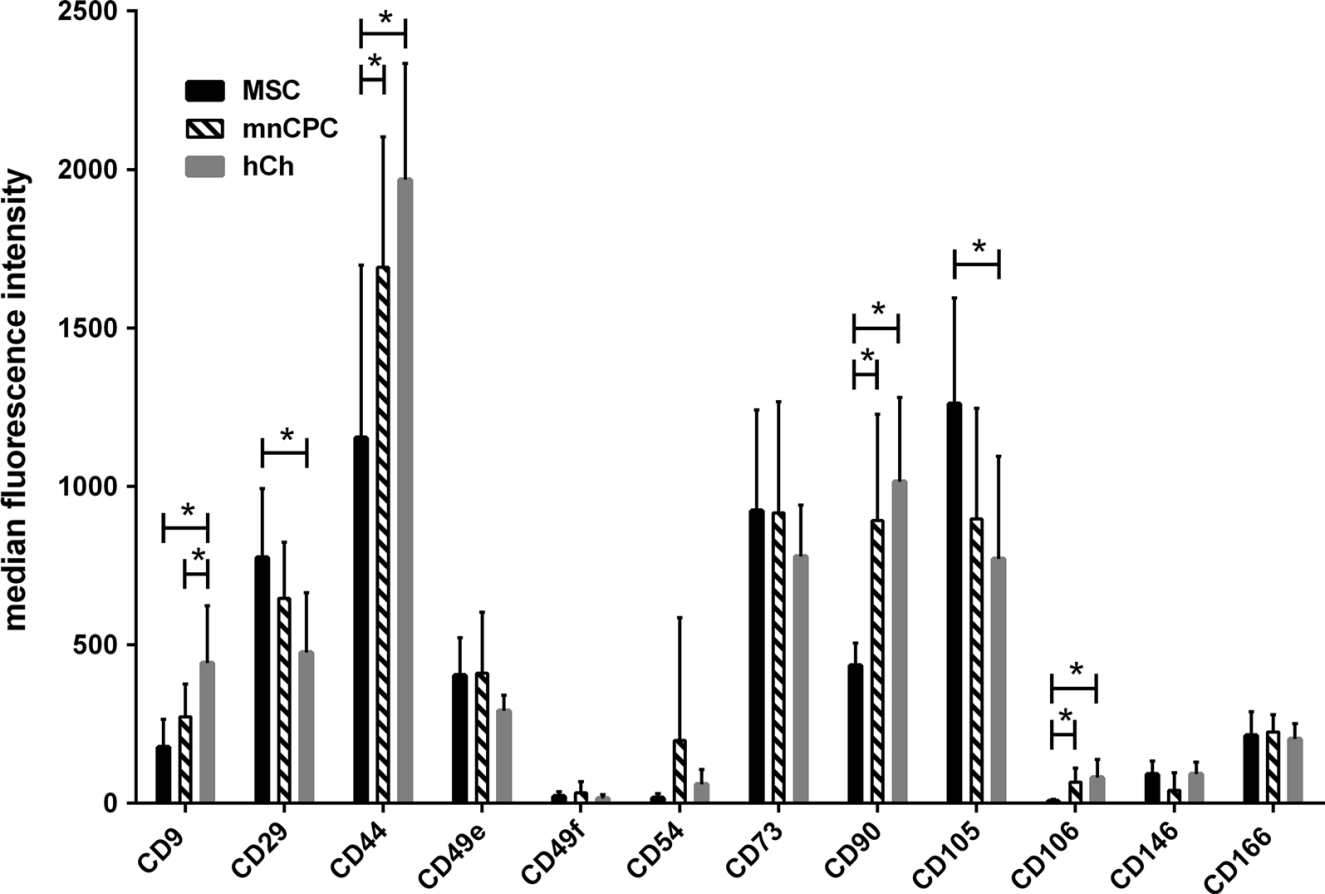
FACS analysis of surface marker expression. The expression of surface markers is given as median fluorescence intensity normalised to the respective isotype control. MSC (*black* n = 9), mnCPC (*black and white stripes* n = 10) and hCh (*dark grey* n = 9). *p ≤ 0.05. For determination of CD166, only 5–9 independent samples of each cell type were available

Several differences were detected in the expression level of the surface markers by the median fluorescence intensity (FI) (mean ± SD) (Fig. 2). HCh demonstrated a significantly higher expression level of CD9 (442.5 ± 181.36) compared to mnCPC (273.2 ± 103.4) and MSC (177.1 ± 88.1). Furthermore, hCh (475.0 ± 189.5) showed a significantly lower expression level of CD29 than MSC (775.6 ± 217.83), while for mnCPC an intermediate expression level (646.2 ± 177.7) was found. The expression of CD44 was significantly higher on hCh (1967.5 ± 366.5) as well as mnCPC (1691.2 ± 411.1) compared to MSC (1152.9 ± 545.1). The FI of CD105 expression revealed a significantly higher expression level on MSC (1260.5 ± 334.33) than on hCh (770.9 ± 324.4), whereas mnCPC (897.2 ± 349.0) showed an intermediate expression, although the differences were not significant. On MSC (6.5 ± 6.2) only a low level of CD106 was expressed, while the expression level for CD106 on mnCPC (67.0 ± 44.2) and hCh (80.8 ± 57.6) was significantly higher. Additionally, MSC (434.3 ± 71.0) expressed significantly less CD90 on each cell compared to mnCPC (892.0 ± 335.8) and hCh (1014.8 ± 265.2). Opposed to the above markers, the expression levels of CD49d, CD49e, CD49f, CD54, CD73, CD166 and CD146 did not reveal any significant differences between the three cell types.

### Differentiation potential of mnCPC lies in between hCh and MSC

**Fig. 3:**
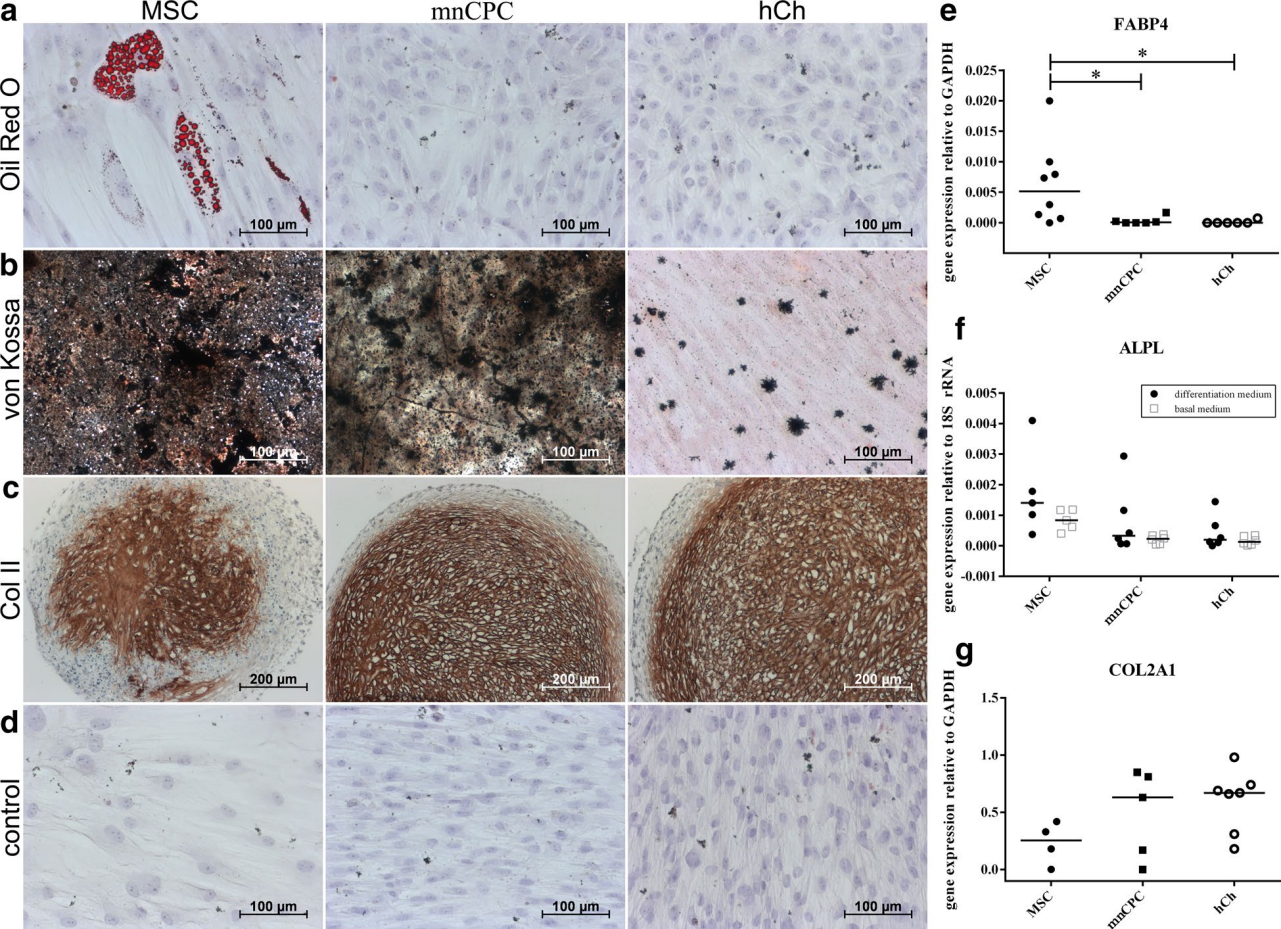
The differentiation potential of mnCPC was compared to hCh and MSC and induced by growth factor supplemented differentiation media over a period of 21 (adipogenic and osteogenic differentiation) and 28 days (chondrogenic differentiation). Only MSC, cultured in adipogenic differentiation medium, exhibited fatty vacuoles positive with Oil red O staining (**a**). Strong calcium deposition—stained with von Kossa stain—was shown for MSC and mnCPC, only small deposits were found in hCh (**b**). In the pellet culture for chondrogenic differentiation, collagen type II was detected in all samples (**c**). All control groups, cultured in basal medium, were negative (**d**). The respective gene expression analysis (**e**–**g**) supported the findings visualised by histological and immunohistochemical staining. *Graphs* show individual values with median, n = 4–8 donors for each cell type, *p ≤ 0.05. Representative images were chosen

To investigate the multilineage differentiation capacity of mnCPC, the cells were seeded either in monolayer (for adipogenic and osteogenic differentiation) or 3D micromass culture (chondrogenic differentiation) and compared to hCh and MSCs. Adipogenesis and osteogenesis were induced for 21 days, chondrogenesis for 28 days. The success of the respective differentiation (Fig. 3) was confirmed by histological and immunohistochemical staining methods as well as gene expression analysis of respective marker genes [fatty acid binding protein 4 (FABP4) for adipogenic, alkaline phosphatase (ALPL) for osteogenic and collagen type II (COL2A1) for chondrogenic differentiation].

During induction of adipogenesis, colonies containing lipid vacuoles positive for oil red O, were observed only in MSC (Fig. 3a), while neither mnCPC nor hCh produced fatty vacuoles. Also FABP4 was not upregulated in mnCPC and hCh as opposed to MSC (Fig. 3e), FABP4 mRNA was not detectable in any of the samples cultured in basal medium.

The detection of black calcium deposits with von Kossa staining, confirmed the osteogenic potential of mnCPC and MSC (Fig. 3b). Clearly the staining intensity and distribution was much stronger in both MSC and mnCPC as opposed to hCh. The expression analysis of ALPL (Fig. 3f), confirmed the staining, although ALPL was also expressed in control samples of all cell types. In these experiments, mnCPC had an intermediate differentiation capacity in between hCh and MSC.

Micromass culture and simultaneous stimulation with soluble chondrogenic factors for 4 weeks induced chondrogenic differentiation in all three cell types MSC, mnCPC and hCh, detectable by a strong positive staining for collagen type II (Fig. 3c) and on gene expression level with the expression of COL2A1 (Fig. 3g). Differentiation of MSC was weaker as opposed to hCh and mnCPC. Pellet culture of all three cell types in basal medium was not possible, due to complete disintegration of the cell pellets after 2 weeks. Therefore no histological stainings can be presented. In the control cells, which were cultured as monolayers in basal medium, no positive staining was observed (Fig. 3d).

### Migratory activity of mnCPC is stimulated by PDGF-BB and IGF-I

**Fig. 4:**
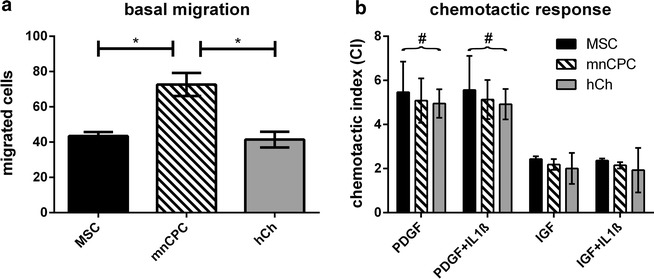
Cell migration and response to chemotactic factors. MnCPC showed a significantly higher basal migration in a Boyden chamber assay compared to MSC and hCh (**a**). All three cell types responded comparably to chemotactic factors (**b**), stimulation with PDGF-BB was always significant compared to basal migration (#). *p ≤ 0.05, n = 3 donors for each cell type

As shown in Fig. 4a, mnCPC revealed a significantly higher basal migratory activity than MSC and hCh. PDGF-BB (10 ng/ml) significantly stimulated the migration in all analyzed cell types whereas IGF-I (100 ng/ml) enhanced migration only in tendency (Fig. 4b). The chemotactic index (CI) was comparable in MSC, mnCPC and hCh. Co-stimulation with IL-1β (1 ng/ml) did not alter PDGF-BB- or IGF-1-induced cell migration. The effect on cell migration was only present in case of a concentration gradient (data not shown), indicating that in all cases site-directed migration (chemotaxis) was induced and not random migration (chemokinesis). To investigate if the differences between mnCPC and hCh in migratory properties are due to donor specific differences, we determined basal migration and PDGF-BB induced CI of mnCPC and hCh of the same donor (n = 3). The results correspond exactly to the results given in Fig. 4 (data not shown).

### MnCPC migrate into DECM and produce cartilage-specific ECM

**Fig. 5:**
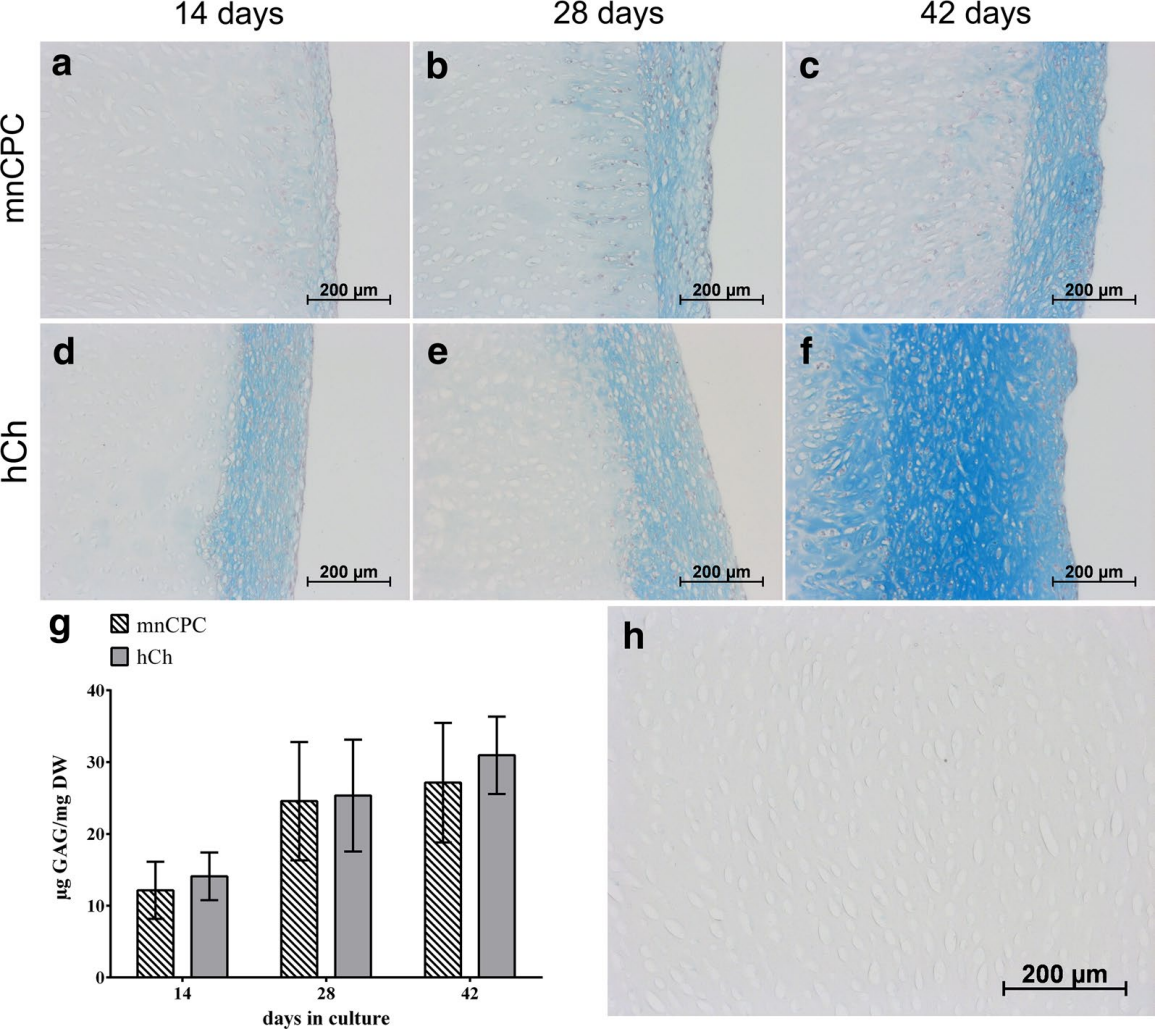
GAG synthesis of mnCPC (**a**–**c**) and hCh (**d**–**f**) on DECM evaluated by alcian blue staining and quantified with DMMB assay (**g**). GAG synthesis was detectable on day 14 for mnCPC (**a**) and hCh (**d**). The GAG synthesis increased clearly from day 14 until day 42 (**c**, **f**). The DECM without cells contained no stainable GAG (**h**). Compared to mnCPC, hCh demonstrated a slightly higher synthesis and accumulation (**g**) of GAG during culture for up to 42 days. GAG content was normalised in respect to cell number and dry weight of unseeded DECM. The *values* represent the mean ± SD with n = 12 replicates

**Fig. 6:**
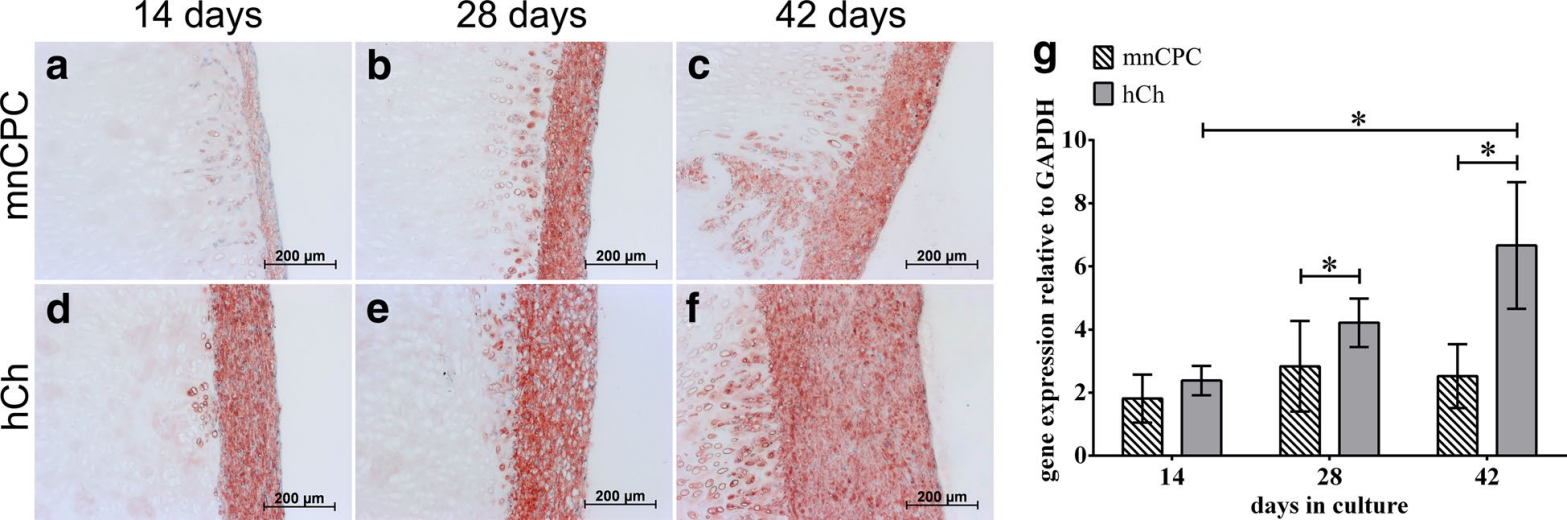
Aggrecan synthesis of mnCPC (**a**–**c**) and hCh (**d**–**f**) on DECM evaluated on protein (**a**–**f**) and gene expression level (**g**). Aggrecan synthesis was detectable on day 14 for mnCPC (**a**) and hCh (**d**). The aggrecan synthesis increased clearly from day 14 until day 42 (**c**, **f**). Compared to mnCPC, hCh demonstrated a significantly higher expression of aggrecan (**g**). The *values* represent the mean ± SD with n = 8 donors for each cell type, *p ≤ 0.05

**Fig. 7:**
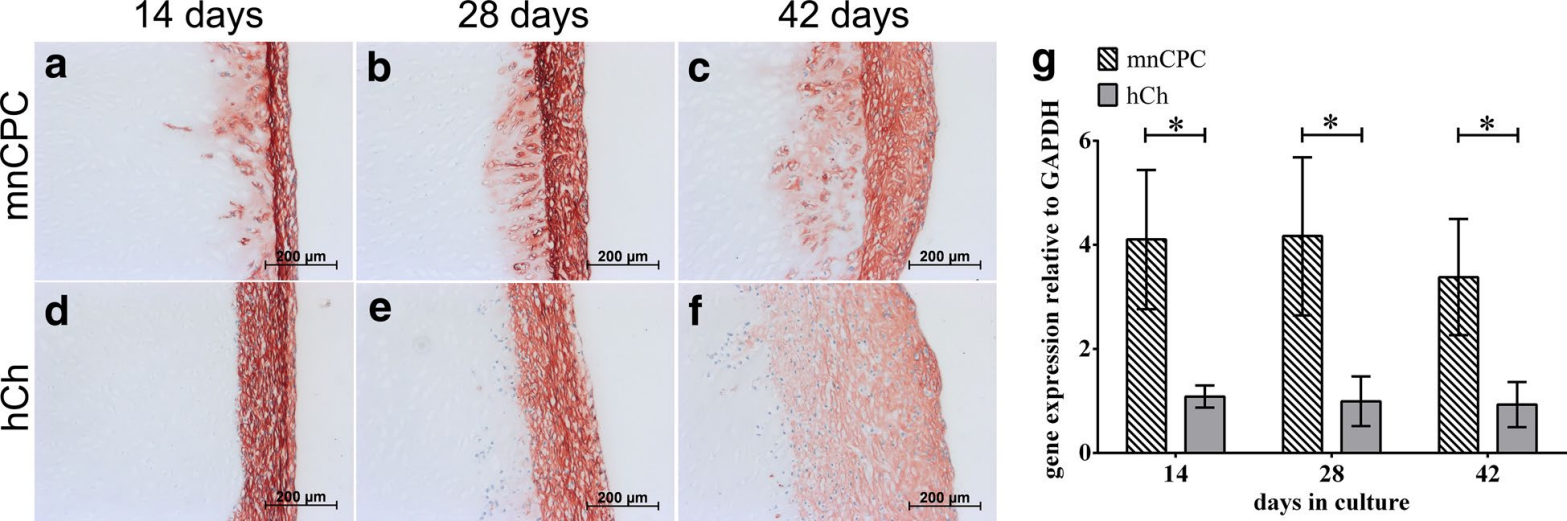
Collagen type I production (**a**–**f**) as well as gene expression (**g**) was detected for mnCPC (**a**–**c**) and hCh (**d**–**f**). On gene expression level (**g**) mnCPC demonstrated a significantly higher collagen type I expression than hCh. The gene expression analysis correlated with the detection of collagen type I on protein level by immunohistochemical staining. The *values* represent the mean ± SD with n = 8 donors for each cell type, *p ≤ 0.05

An increasing accumulation of sulphated glycosaminoglycans (GAG) in the superficial zones of the scaffolds as well as in the cell layers on the construct surfaces (Fig. 5) was demonstrated by an increasing alcian blue staining during 3D culture of DECM seeded with mnCPC and hCh. For both cell types, GAG deposition increased visibly starting from day 14 to day 42 and spread deeper into the scaffolds until day 42. The GAG quantification on protein level with dimethylmethylene blue (DMMB) (Fig. 5g) supported the increasing neo-synthesis and accumulation of GAG. Due to the chemical production process, the DECM scaffold itself did not contain any stainable GAG (Fig. 5h). The intensity of the aggrecan staining (Fig. 6) indicated that hCh accumulate clearly more aggrecan than mnCPC, while at the same time the distribution of cell nuclei evidenced that both, mnCPC and hCh, migrated into the matrix within the first 2 weeks of 3D culture in a comparable manner. Active cell migration of mnCPC and hCh was demonstrated by the presence of small channels which were formed in many regions of the scaffold surface (Figs. 6, 7). Along these channels and in close vicinity to migrating mnCPC and hCh, aggrecan neosynthesis was detected.

**Fig. 8:**
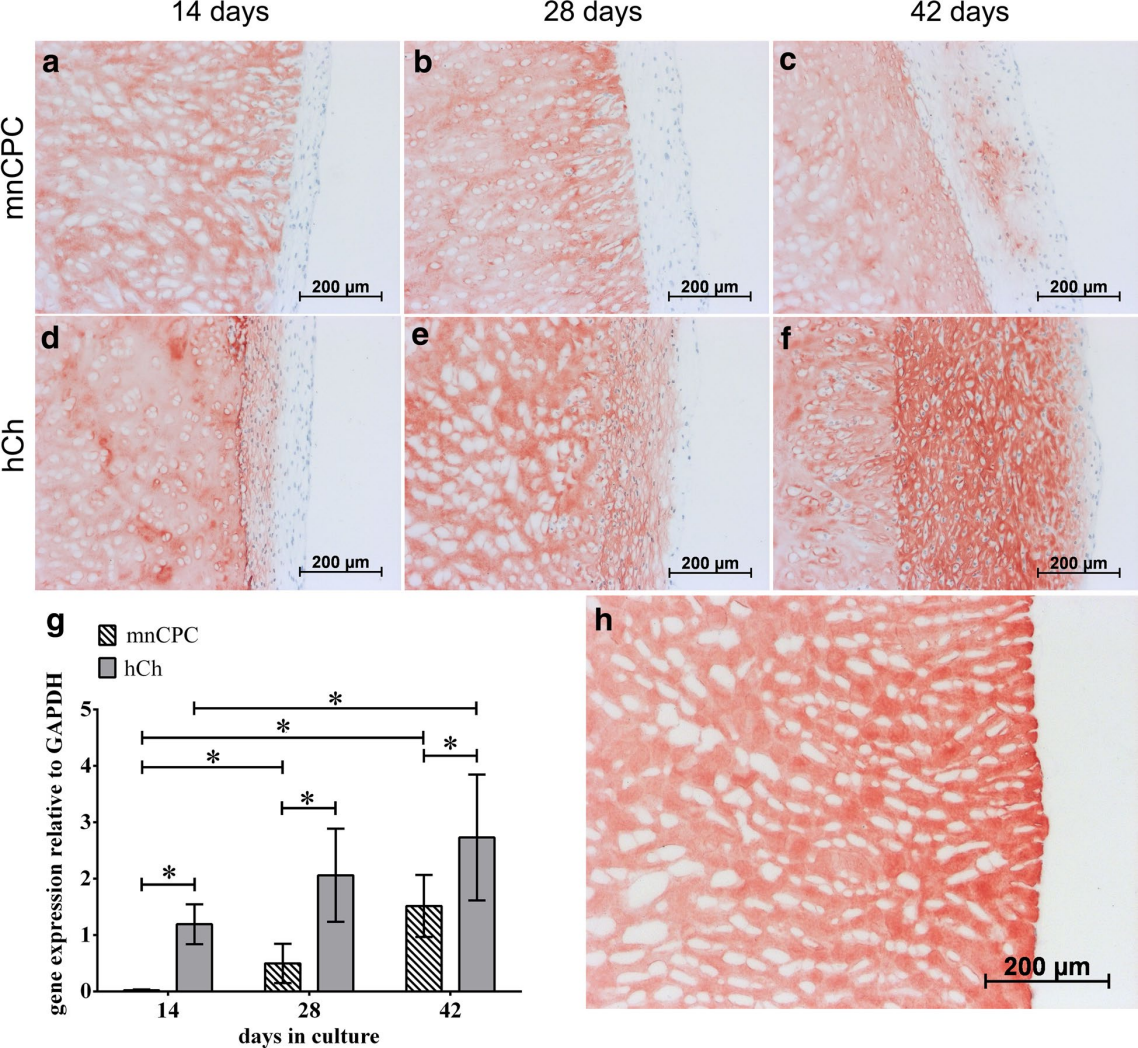
Immunohistochemical staining of collagen type II (**a**–**f**) as well as gene expression analysis (**g**) reflected the significantly higher collagen type II synthesis of hCh compared to mnCPC. On day 14, hCh demonstrated a slight collagen type II synthesis in the superficial cell layer on the construct surface (**d**) while mnCPC exhibited no collagen type II production. During the 3D culture for up to 42 days, collagen type II synthesis of hCh increased (**d**–**f**). This increase was additionally detected on gene expression level. mnCPC (**a**–**c**) demonstrated a slight collagen type II synthesis starting from day 28 (**b**, **g**) and increasing until day 42 (**c**, **g**). The scaffold matrix consists of collagen type II and reacted strongly with the collagen type II antibody (Fig. 6h). The *values* represent the mean ± SD with n = 8 donors for each cell type, *p ≤ 0.05

The capacity of mnCPC to synthesize ECM proteins in comparable amounts as hCh was furthermore examined by immunohistochemical staining for collagen types I (Fig. 7) and II (Fig. 8). Production of collagen type I was detectable within the tight layer of hCh and mnCPC on the scaffold surface. Compared to hCh, mnCPC expressed significantly higher amounts of COL1A1 mRNA (Fig. 7g).

Collagen type II synthesis was detected on the surface of DECM constructs seeded both, with mnCPC as well as hCh. The collagen type II *de novo* synthesis of hCh (Fig. 8) increased significantly during progressing 3D culture (Fig. 8g). Scaffolds seeded with mnCPC demonstrated no collagen type II synthesis at day 14 and 28. The staining for cartilage specific collagen type II was first detectable on day 42. Due to the composition of the DECM scaffolds, newly synthesized collagen type II was not distinguishable from original collagen type II present in DECM. The scaffold matrix consists of chemically treated hyaline porcine cartilage and therefore reacts strongly with the collagen type II antibody (Fig. 8h). On gene expression level a significantly higher expression of COL2A1 was observed in hCh as opposed to mnCPC. Still, gene expression of COL2A1 increased significantly with increasing cultivation time in both cell types. Cultivation of MSC on DECM resulted in cell attachment on the surface, but did not show proliferation and matrix synthesis even under chondrogenic stimulation. Therefore, no cell culture results could be obtained with MSCs.

### MnCPC have a stronger MMP-9 activation than hCh

**Fig. 9:**
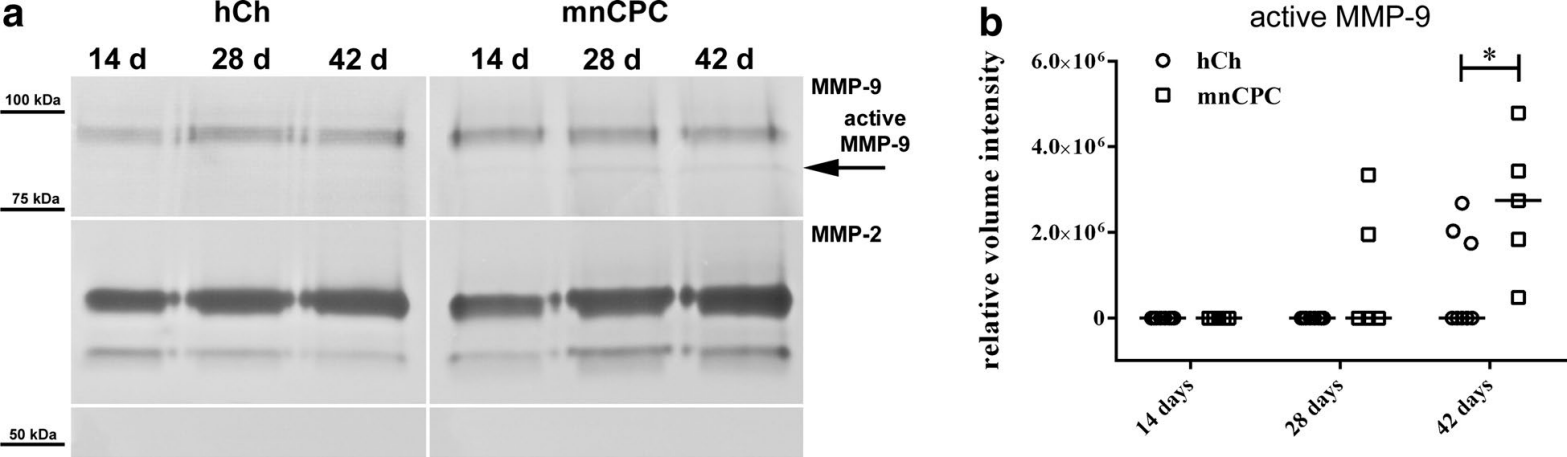
Gelatin zymography demonstrated a higher secretion of active MMP-9 of mnCPC as opposed to hCh (hCh n = 8 donors, mnCPC n = 5 donors) after 42 days (**a**–**b**). Detection of pro-MMP 9 revealed a similar expression level at all points (**a**). For MMP-2 no difference was observable neither for the active nor the inactive form of the endopeptidase over time (**a**). Image is composed of several gel images separated by *white lines*. To normalize the measured intensities of the samples on different gels and to correct differences between the runs, a standard sample was carried along on every gel. All values were normalized to the detected intensity of the standard sample. *Graph* shows individual values with median, Mann–Whitney-U comparison, *p ≤ 0.05

The presence of the gelatinases A [matrix metalloproteinase 2 (MMP-2)] and B (MMP-9) was assessed by gelatin zymography, because it is known that these gelatinases are involved in eliminating partially degraded collagens [36, 37]. Culture supernatants of the DECM scaffolds seeded with mnCPC or hCh cultured in chondrocyte differentiation medium for 14, 28 and 42 days were loaded onto gels (Fig. 9). As depicted in Fig. 9a, 14 days after seeding no active MMP-9 was detected. A significantly higher protein expression of active MMP-9 at day 42 was detected in mnCPC as compared to hCh (Fig. 9b). Protein expression of proMMP-2 as well as active MMP-2 (Fig. 9a) was comparable in hCh and mnCPC.

## Discussion

The aim of this study was to assess whether healthy adult human septal cartilage harbors a subpopulation of cells with migrative and progenitor cell features (mnCPC) as has been demonstrated previously in osteoarthritic joint cartilage [30]. Furthermore, these cells should be amplified and characterized in some detail. Within this study, we were able to isolate mnCPC from healthy adult human septal cartilage and demonstrated that this cell population was able to differentiate into osteogenic and chondrogenic cell types. Compared to MSC and hCh, mnCPC exhibited a different surface marker profile, which might be positioned between hCh and MSC. To assess the potential for in vivo mobilization and thus a potential role in cartilage regeneration procedures, we seeded DECM scaffolds with mnCPC and hCh and cultured them up to 42 days in vitro. Expression and production of cartilage specific proteins, synthesis rate of GAG and migration characteristics confirmed that mnCPC are comparable to hCh.

In contrast to two previous studies on nasoseptal progenitor cells [38, 39], which isolated CPC from nasal septum cartilage by enzymatic digestion, we hypothesized that mnCPC grow out from small cartilage biopsies. This enabled us to utilize migrative properties as a selection criterion for this specific cell population. On average, colonies of cells which had migrated out of cartilage pieces were confluent after 16 days in culture, which is comparable to previously reported data for osteoarthritic articular CPC [30, 31].

Phenotypically, mnCPC combined morphologic characteristics of MSC and hCh. After 5 passages in monolayer culture, hCh maintained their round shape and did not exhibit the elongated cell morphology of mnCPC of passage 1. MnCPC showed proliferation activity up to 20 passages and were able to form significantly more colonies than hCh in vitro demonstrating their clonogenicity. Thus there is some morphological evidence that differences in differentiation capacity and surface markers are not caused by different cultivation time of mnCPC and hCh, which is caused by the different isolation procedures and thus unavoidable.

The surface marker profile of mnCPC was very similar to progenitor cells obtained by outgrowth from human osteoarthritic [31] or healthy bovine articular cartilage [40]. CD29 is often used in combination as a surface marker for cells with mesenchymal stem cell characteristics [21] and was expressed by MSC at the highest level in our experiments. In agreement with a recent study on CPC derived from bovine knee joints [40], mnCPC exhibited an enhanced expression of CD29 compared to hCh.

MnCPC expressed CD44, the major receptor for hyaluronan, at a significantly higher level than MSC, but at the same time significantly lower than differentiated hCh. Grogan et al. [41] showed that high-chondrogenic-capacity cells demonstrated an increased expression of CD44 and superior capacity for cartilage tissue formation. The high CD44 expression emphasizes the enhanced chondrogenic potential of mnCPC as opposed to MSC. In accordance with the results of Seol et al. [40] which were obtained from articular CPC, mnCPC showed a higher expression of CD105 compared to hCh, while CD90 expression of mnCPC was lower when compared to hCh.

In our experiments only MSC from bone marrow differentiated into adipogenic cells, as demonstrated by oil red O stain. MnCPC differentiated in the osteogenic and chondrogenic lineage, while hCh only weakly deposited Ca^2+^ in the von Kossa stain. FABP4, which is a late marker for adipogenesis [42, 43] was absent in mnCPC and hCh and thus confirmed the results of the oil red O stains.

The literature provides plausible explanations for the bipotential differentiation capacity of mnCPC to differentiate only into the chondrogenic and osteogenic directions. Shafiee et al. observed [38] that only one colony of their nasal septal progenitor cells differentiated in all lineages indicating a significant heterogeneity of this cell population. MSC preparations from bone marrow always contain a multiplicity of clones with tripotent, bipotent and unipotent characteristics [44]. Also Amaral et al. were not able to induce adipogenic differentiation in the progenitor cells they isolated from human nasal cartilage [39]. This suggests that their enzymatically isolated cells might be a similar subpopulation of cells as mnCPC derived by outgrowth.

In both, mnCPC and MSC, von Kossa stain showed strong Ca^2+^ deposition, while hCh revealed only single cells containing Ca^2+^ deposits. The gene expression of the commonly used marker ALPL [21, 45] reflects the characteristics of MSC and mnCPC to undergo osteogenic differentiation and confirms the results of visualized Ca^2+^ accumulation. Thus, there is evidence that mnCPC are less differentiated compared to fully differentiated hCh.

Under chondrogenic induction in micromass culture, all three cell types expressed chondrogenic matrix components. The synthesis of collagen type II started in the center and was stronger for mnCPC and hCh than for MSC. This observation was confirmed on gene expression level. The outer layers of the pellets were positive for collagen type I, which in culture is often considered to indicate the existence of fibrocartilage [46]. Williams and coworkers suggest that a time dependent collagen expression starting with collagen type I followed by collagen type II indicates a developmental process of maturing cells [46, 47]. A zoned co-localization of collagen type I and II was described at articular surfaces between interzone cells and perichondrium in the developing limb [48].

Similar observation were made on seeded DECM. Collagen type I was detectable during the whole observation time by both cell types, and was always significantly higher expressed by mnCPC, underlining that mnCPC are not as differentiated as hCh are. HCh started to synthesize collagen type II in the inner layer of seeded cells after 14 days, mnCPC produced collagen type II only after 42 days, indicating that maturation of mnCPC takes several weeks of time. Chondrocytes with high migratory activity, isolated from injured articular cartilage, showed a similar under-expression of the cartilage specific genes such as collagen type II and aggrecan compared to normal chondrocytes [40].

Comparable to articular CPC [30, 31], we detected a high migratory ability of mnCPC in a Boyden chamber assay, exceeding the migration activity of MSC and nasal hCh under basal and growth factor stimulated conditions. A migratory response of articular hCh to IGF-I and of MSC to PDGF-BB and IGF-I has already been described [33, 34, 49]. The analyzed cell types in our study revealed comparable chemotactic indices (CIs) indicating a similar potency of stimulation on the basis of a higher basal migration potential of mnCPC. This finding goes in line with a faster repopulation of mnCPC-seeded DECM compared to hCh. The migratory activity of mnCPC was comparable to that found for articular CPC concerning IGF-I-stimulation but was higher concerning basal migration and PDGF-BB induced response [30]. Due to this high migratory potency, mnCPC may be a potent cell source for in situ regeneration keeping in mind that cell recruitment could be further enhanced by local application of PDGF-BB and IGF-I. A surgical strategy of cartilage defect reconstruction always implicates the induction of an inflammatory response. The migratory activity of articular CPC from patients with osteoarthritis was strongly inhibited by the pro-inflammatory cytokine IL-1β [30]. However, neither mnCPC nor nasal hCh nor MSC were impaired by IL-1β concerning stimulated migration, as has already been described for bone-marrow-derived MSC [50]. So far, it is not known whether this difference can be attributed to the osteoarthritis disease process or to primary characteristics of these cell populations. This remains to be elucidated.

The presented results as well as unpublished results of in vivo studies in a rabbit model suggest that mnCPC are not the cell population of primary choice for classic tissue engineering procedures due to the high overexpression of collagen type I. But the interesting observation is the presence of a chondrogenic cell subpopulation in nasoseptal cartilage with progenitor cell and migratory properties distinguishable from nasoseptal chondrocytes and bone-marrow-derived MSC. An endogenous cell source with high migratory activity as well as chondrogenic potential such as the presented mnCPCs provide potential for in vivo cartilage regeneration strategies and lateral integration of implant matrices in vivo.

Since the activity of matrix degrading enzymes is necessary for cell migration in tissues we analyzed MMP-2 and MMP-9 secretion and activity in supernatants of seeded DECM by gelatin zymography. Adult articular chondrocytes constitutively express MMP-2 suggesting this MMP is involved in physiological collagen turnover [51, 52]. MnCPC and hCh secreted MMP-2, which plays a role in eliminating partially degraded collagens [36], in a comparable manner. Active MMP-9 was detectable in supernatants of mnCPC-seeded but not hCH-seeded DECM after 42 days of culture. MMP-9 has been shown to digest ECM [51] and to cleave products of collagen type I and II proteolysis often associated with osteoarthritis [53]. We hypothesize that in our experiments, MMP-9 is involved in the digestion of ECM which results in the formation of channels enabling cells to migrate actively into the collagenous matrix. According to Heissig et al., stem and progenitor cells require MMP-9 to differentiate and to get mobilized from their quiescent state [54]. This finding goes in line with our current observations and is a prerequisite for in situ tissue engineering.

## Conclusions

In summary, we were able to demonstrate the presence of migratory active chondrocytes with progenitor cell features in adult human nasal cartilage which share important characteristics with bone marrow-derived MSC and nasal hCh. MnCPC are potential target cells for in vivo regeneration strategies of the nasal septum. The limited availability of fully differentiated chondrocytes and the complexity of combining cells and biomaterials in vitro could possibly be circumvented by such a strategy. Responsiveness of mnCPC to PDGF-BB and IGF-1 might be exploited to enhance cell recruitment by local application of these factors.

## Methods

### Isolation of hCh and mnCPC

Donors of nasoseptal cartilage underwent septoplasties or septorhinoplasties in the Department of Otorhinolaryngology, Head and Neck Surgery, University Medical Center Ulm. The age of donors for mnCPC ranged from 22 to 53 years with an average age of 33.4 ± 10.8 years (*n* = 8, gender ratio female/male 1/7) and for hCh from 18 to 41 years with an average age of 23.2 ± 6.3 years (*n* = 12, gender ratio female/male 4/8). Cartilage harvesting was approved by the University of Ulm Ethical Committee (No.: 152/08). The superficial cartilage layer with the remaining adjacent perichondrium was cut off with a scalpel and the purified cartilage samples were cut into 1 × 1 mm pieces.

For isolation of mnCPCs, the minced cartilage was transferred to 25 cm^2^ culture flasks containing basal medium (DMEM/Ham’s F12 (1:1), 10 % fetal bovine serum (FBS), 1 % penicillin/streptomycin, all purchased from Biochrom, Berlin, Germany). Seven days after the initial cartilage transfer, cells started to grow out. When reaching 80–90 % confluence, cells were trypsinized.

To isolate hCh, the minced cartilage was digested in basal medium containing 0.3 % collagenase type II (Worthington, Lakewood, NJ, USA) at 37 °C over night.

After isolation, hCh and mnCPC were amplified in monolayer culture with basal medium, seeded at an initial cell density of 5 × 10^3^ cells cm^−2^. When reaching 80–90 % confluence, cells were trypsinized and cryopreserved.

### Harvesting and culture of human bone marrow derived MSC

Bone marrow aspirates from five human donors were obtained from Lonza (Basel, Switzerland). Donor age ranged from 21 to 41 years with an average age of 32.6 ± 7.8 (*n* = 5, gender ratio female/male 1/4). 5 mL of each bone marrow aspirate was seeded in 150 cm^2^ cell culture flasks and covered with DXX Medium (Gibco, Carlsbad, CA, USA) supplemented with 1 % l-glutamine, 1 % penicillin/streptomycin, 10 % FBS and sodium-pyruvate (all purchased from Biochrom). After 4 days, the cell culture supernatant containing the hematopoietic cell fraction of bone marrow was removed and MSC were isolated by plastic adherence. When cells reached confluence, cells were detached by trypsinization and cryopreserved until further use.

### Clonogenic assay and growth properties

To examine the colony-forming capacitiy and clonogenicity of mnCPC and hCh, cells were seeded in passage 2 at low densities with an initial cell number of 100 cells per 35 mm well. After 8 days, colonies were stained with 0.5 % crystal violet in methanol and counted under a light microscope. Only colonies containing more than 50 cells were scored as colony forming unit and CFE was calculated as the percentage of plated cells that formed colonies. Concerning MSC, other conditions had to be chosen for the CFU assay because of their high proliferation rate. A CFE of 81.3 ± 12.8 % was detected after seeding 25 cells per 35 mm well and 14 d of cultivation. As this assay is not comparable to the CFU assay performed with mnCPC and hCh, the results were not presented in the results section.

Cumulative population doubling level (PDL) at each subcultivation of mnCPC and hCh (n = 3) was calculated using the following equation: PD = log(N/N_0_)/log2, where N = final cell count and N_0_ = initial cell number. The population doubling (PD) was then added to the previous PD, to yield the PDL. The end of the replicative lifespan was reached when cells stopped to proliferate and cell numbers remained constant or decreased.

### Differentiation assays

After thawing and cultivation of all three cell types in the respective basal medium until 80 % confluence, cells were trypsinized (passage 2), seeded in 6-well plates and cultivated in differentiation media or basal media as control for up to 21 days for adipogenic and osteogenic differentiation. The initial cell density was 3.15 × 10^4^ cells cm^−2^ in MSC adipogenic differentiation medium (PromoCell, Heidelberg, Germany). For osteogenic differentiation, cells were seeded with a density of 3.6 × 10^3^ cells cm^−2^ in StemMACS OsteoDiff Media (Miltenyi Biotec, Bergisch Gladbach, Germany). For chondrogenic differentiation in micromass culture, 2 × 10^5^ cells were pelleted by centrifugation and cultivated in StemMACS ChondroDiff Media (Miltenyi Biotec) for 28 days. All media were supplemented with 1 % penicillin/streptomycin (Biochrom). For all differentiations, parallel attempts were seeded for subsequent gene expression analysis.

### Decellularized extracellular cartilage matrices

Sterile, disc shaped scaffolds made of DECM with a diameter of 5 mm and a height of 1 mm were prepared at the Institute of Bioprocess Engineering of the University of Erlangen as described before [35].

### Seeding and 3D culture on DECM

For scaffold seeding, hCh and mnCPC were thawed and cultured in monolayer until 80–90 % confluence. Scaffolds were seeded either with 1.0 × 10^6^ hCh or mnCPC (passage 2) per scaffold as published previously [11]. Seeded scaffolds were cultured in StemMACS ChondroDiff Media (Miltenyi Biotec) supplemented with 0.5 % gentamycin (Biochrom) for up to 42 days in a humidified atmosphere with 5 % CO_2_ at 37 °C. Medium was changed twice a week. Scaffolds were analyzed on days 14, 28 and 42.

### Histological analyses

Seeded scaffolds and pellets were fixed in 3.5–3.7 % neutral buffered formalin solution (Fischar, Saarbruecken, Germany), embedded in paraffin, sectioned at 4 µm and incubated at 56 °C over night. Sections of seeded scaffolds were rehydrated and stained with alcian blue to detect acidic sulphated proteoglycans.

Differentiation was confirmed with von Kossa stain and Oil Red O stain. Both were performed as previously described [28].

### Immunohistochemical detection of collagen type I, collagen type II and aggrecan

For immunohistochemical visualization of collagen type II and aggrecan, deparaffinized and rehydrated sections were treated as already described [55].

To detect collagen type I, deparaffinized and rehydrated sections were treated with 1 mg mL^−1^ Pepsin in 0.5 M acetic acid for 2 h at 37 °C. Endogenous peroxidase was blocked and an additional blocking step with Protein Block-serum-free-(DAKO) applied for 30 min. The primary antibody (ab34710, Abcam, UK) was diluted 1:4000, incubated on the sections at 4 °C over night and visualized using the LSAB + System-HRP (DAKO). Nuclei were counterstained with haematoxylin.

### Quantitative assessment of DNA and GAG

DNA content as a measure for number of cells within the scaffold matrix, were estimated with the Hoechst assay according to Kim et al. [56]. The amount of GAG was quantified spectrophotometrically using the DMMB-assay as described elsewhere [57]. Both assays were adjusted with slight modifications as published recently [11, 35].

### Quantitative real-time PCR

Seeded scaffolds were snap-frozen after 14, 28 and 42 days (each *n* = 8) and homogenized in RLT-buffer (Qiagen, Hilden, Germany) with a tissue lyser (Qiagen) for 5 min at 50 Hz. RNA was isolated and purified using the RNeasy Mini kit according to the manufacturer’s instructions (Qiagen), including on-column DNAse treatment. RNA of monolayer cultures and of micromass culture were isolated immediately with the RNeasy Mini kit (Qiagen).

Subsequently, 100 ng of total RNA were reversely transcribed with the QuantiTect Reverse Transcription Kit (Qiagen). Real-time PCR using a LightCycler 2.0 (Roche, Basel, Switzerland) was performed with 2 μL of each cDNA sample in duplicate. The detected genes, primers and used probes (Universal Probe Library, Roche) are shown in Table 1. After an initial denaturation at 95 °C for 10 min, amplification was performed in 45 cycles: 95 °C for 10 s, followed by 60 °C for 20 s and 72 °C for 1 s. The relative expression level of the housekeeping gene glyceraldehyde-3-phosphate dehydrogenase was used to normalize samples. Relative quantification of marker gene expression was calculated according to 2^−∆Ct^, at which ΔCt = Ct of the target—Ct of the reference.

**Table 1:** Analysed genes and information about sequences of primers, product size and used probes for real-time PCR

Gene description	Gene name	Primer left (5′–3′)	Primer right (5′–3′)	Product size (bp)	UPL probe
Glyceraldehyd-3-phosphate dehydrogenase	GAPDH	gctctctgctcctcctgttc	acgaccaaatccgttgactc	115	# 60
Aggrecan	ACAN	tgcagctgtcactgtagaaactt	atagcaggggatggtgagg	112	# 79
Collagen, type I, alpha 1	COL1A1	atgttcagctttgtggacctc	ctgtacgcaggtgattggtg	126	# 15
Collagen, type II, alpha 1	COL2A1	ccctggtcttggtggaaac	tccttgcattactcccaactg	88	# 19
Fatty acid binding protein 4	FABP4	cctttaaaaatactgagatttccttca	ggacacccccatctaaggtt	105	# 72

For quantitative real-time PCR-analysis of ALPL, the TaqMan^®^ Gene Expression Assay Hs01029144_m1 was used with TaqMan^®^ Gene Expression Master Mix in a StepOnePlusTM Real-Time PCR System (all from Applied Biosystems, Darmstadt, Germany). The ALPL expression level was normalized to 18S rRNA as described [58].

### Gelatin zymography

Culture supernatants of scaffolds seeded with mnCPC (*n* = 5 donors) and hCh (*n* = 8 donors) were collected at day 14, 28 and 42 and stored at −20 °C. Due to regular medium change, each supernatant was incubated with scaffolds for 3 days. Expression as well as activity of MMP-2 (= gelatinase A) and MMP-9 (= gelatinase B) were determined by gelatin zymography using 2 mg/mL of gelatin (Merck) as a substrate in 10 % SDS–polyacrylamide gels. For detection of MMP-2, supernatant was diluted 1:15 with PBS, MMP-9 was detected in undiluted supernatants. The culture supernatants were loaded on the zymography gels and proteins were separated electrophoretically for 3 h at 4 °C. The gels were washed with Zymogram renaturation buffer (BioRad, Hercules, CA, USA) for 30 min. Bands developed during 20 h incubation in Zymogram development buffer (BioRad) at 37 °C. Subsequently, gels were stained for 1 h with 0.34 % Coomassie Brilliant Blue G-250 (Carl Roth, Karlsruhe, Germany) in 10 % acetic acid and 40 % methanol and destained for 3 h in a 20 % acetic acid/80 % methanol solution. All steps of renaturation, development and staining were done under slight agitation. To normalize the measured intensities of the samples on different gels and to correct differences between the runs, a standard sample was carried along on every gel. All values were normalized to the detected intensity of the standard sample. Each value was normalized to its corresponding mean cell number on the scaffold as determined by Hoechst assay (see “Quantitative assessment of DNA” section).

### Chemotaxis assay

Cell migration was analyzed by a modified Boyden chamber assay, using a 48-well microchemotaxis chamber (NeuroProbe Inc., Baltimore, MD, USA) with polycarbonate filters with 8 μm pores (NeuroProbe Inc.) between the lower well containing the chemotactic factor and the upper well containing the cells. Recombinant human PDGF-BB (BioLegend, Fell, Germany, 10 ng/ml), rhIGF-1 (Biomol, Hamburg, Germany, 100 ng/ml) and IL-1β (tebu-bio, Offenbach, Germany, 1 ng/ml) were diluted in serum-free DMEM, filled into the lower compartment of the chemotaxis chamber and covered with the chemotaxis filter. The upper wells were loaded with 1 × 10^4^ cells, suspended in serum-free DMEM and incubated for 4 h. Non-migrated cells were removed and migrated cells on the lower side were fixed with 4 % formaldehyde, stained with Giemsa solution (Merck, Darmstadt, Germany) and counted. DMEM in the lower well served as a negative control (basal migration) for each experiment. To distinguish chemotaxis from undirected chemokinesis migration analyses with the growth factors were performed in the presence and absence of a concentration gradient. The CI was determined as the average number of migrated cells in stimulated wells divided by the average number of migrated cells in control wells.

### Analysis of surface marker expression

Differences in surface marker composition of bone marrow derived MSC, hCh and mnCPC were comparatively examined by flow cytometric analysis (FACS). When reaching 80 % confluence, each cell type was harvested by trypsinization (passage 2). Cells were washed twice with PBS containing 1 % FBS (Biochrom) and 0.02 % sodium azide (Sigma-Aldrich). The cells were incubated for 30 min in ice cold blocking buffer containing specific fluorescein isothiocyanate (FITC)- or phycoerythrin (PE)-labelled mouse anti-human monoclonal antibodies. The following antibodies were used for the detection of human surface antigens: CD9, CD45 and CD90 FITC-labelled, CD29, CD31, CD34, CD44, CD49d, CD49e, CD49f (rat IgG2), CD54, CD73, CD105, CD106, CD133/1, CD133/2, CD146 and CD166 PE-labelled. In all experiments, the respective FITC- or PE-labelled non-immune isotype-matched antibodies were used as negative controls.

Surface marker composition was analyzed on a FACScan flow cytometer with dual-laser technology and CELLQuest software V2 (Becton–Dickinson, Franklin Lakes, NJ, USA). For each antibody 1 × 10^4^ cells of each cell type were used. Dead cells were excluded by propidium iodide (Sigma-Aldrich) staining. Cells were gated on forward and side scatter to exclude debris and cell aggregates. FI emitted by the dye-conjugated specific antibody bound to the antigen was determined and normalized to the median FI emitted by cells stained with respective isotype.

CD133/1, CD133/2 were obtained from Miltenyi Biotec, CD34 from Invitrogen (Carlsbad, CA, USA), CD105 from R&D Systems (Minneapolis, MN, USA), CD9, CD31 and CD106 from BioLegend (San Diego, CA, USA). All other antibodies and the isotype controls (FITC mouse IgG1, PE mouse IgG1, PE rat IgG2) were provided by Becton–Dickinson.

### Statistical evaluation

Statistical analysis was performed with SigmaPlot^®^ 11.2 software (Systat Software GmbH, Erkrath, Germany) and GraphPad Prism version 6.03 for Windows (GraphPad Software, San Diego, CA, USA). Statistical significance was assessed using Kruskal–Wallis one-way analysis of variance followed by the Mann–Whitney U test. Cell migration, FACS experiments and PDT were analyzed with one-way and two-way ANOVA and Bonferroni multiple comparison post-test. For all statistical analyses, differences were considered statistically significant at *p* ≤ 0.05. Data are presented as mean values ± standard deviation of the mean (SD) or plotted as single values with a line indicating the median.
